# Produce from Africa’s Gardens: Potential for Leafy Vegetable and Fruit Fermentations

**DOI:** 10.3389/fmicb.2016.00981

**Published:** 2016-07-05

**Authors:** Folarin A. Oguntoyinbo, Vincenzina Fusco, Gyu-Sung Cho, Jan Kabisch, Horst Neve, Wilhelm Bockelmann, Melanie Huch, Lara Frommherz, Bernhard Trierweiler, Biserka Becker, Nabil Benomar, Antonio Gálvez, Hikmate Abriouel, Wilhelm H. Holzapfel, Charles M. A. P. Franz

**Affiliations:** ^1^Department of Microbiology, Faculty of Science, University of Lagos, LagosNigeria; ^2^Department of Microbiology and Biotechnology, Max Rubner-Institut, KielGermany; ^3^Institute of Sciences of Food Production, National Research Council of Italy, BariItaly; ^4^Department of Safety and Quality of Fruit and Vegetables, Max Rubner-Institut, KarlsruheGermany; ^5^Área de Microbiología, Departamento de Ciencias de la Salud, Facultad de Ciencias Experimentales, Universidad de Jaén, JaénSpain; ^6^Advanced Green Energy and Environment Institute, Handong Global University, PohangSouth Korea

**Keywords:** horticulture, postharvest, fermentation, food security

## Abstract

A rich variety of indigenous fruits and vegetables grow in Africa, which contribute to the nutrition and health of Africa’s populations. Fruits and vegetables have high moisture and are thus inherently prone to accelerated spoilage. Food fermentation still plays a major role in combating food spoilage and foodborne diseases that are prevalent in many of Africa’s resource disadvantaged regions. Lactic acid fermentation is probably the oldest and best-accepted food processing method among the African people, and is largely a home-based process. Fermentation of leafy vegetables and fruits is, however, underutilized in Africa, although such fermented products could contribute toward improving nutrition and food security in this continent, where many are still malnourished and suffer from hidden hunger. Fermentation of leafy vegetables and fruits may not only improve safety and prolong shelf life, but may also enhance the availability of some trace minerals, vitamins and anti-oxidants. Cassava, cow-peas, amaranth, African nightshade, and spider plant leaves have a potential for fermentation, as do various fruits for the production of vinegars or fruit beers and wines. What is needed to accelerate efforts for production of fermented leaves and vegetables is the development of fermentation protocols, training of personnel and scale-up of production methods. Furthermore, suitable starter cultures need to be developed and produced to guarantee the success of the fermentations.

## Introduction

Statistics show that hunger is still a dramatic problem facing humanity and that nearly 795 million people do not have enough food^1^ (Burchi et al., 2011). Hunger as based on caloric deficits is, however, only part of the story, as many of the hungry have access to the minimal required amount of calories, but are deficient in one or more micronutrients. Micronutrient deficiencies are the so-called ‘hidden-hunger’ and affect approximately 2 billion people worldwide (Burchi et al., 2011), with the majority of people occurring on the African continent and the Indian subcontinent (Muthayya et al., 2013). Worldwide, malnutrition is estimated to contribute to more than one third of all child deaths, although it is rarely listed as the direct cause (Bain et al., 2013). In 2013, an estimated 6.3 million children under the age of five died, 2.9 million of these in the WHO Africa region (WHO, 2013).

Dietary micronutrient deficiencies include calcium, copper, iron, iodine, magnesium, selenium, zinc, and/or vitamin A deficiency (Bain et al., 2013; Joy et al., 2014). Micronutrient deficiencies have detrimental effects on children growth and development, and the most common and clinically significant micronutrient deficiencies in children and childbearing women include deficiencies in iron iodine, zinc and vitamin A (Bain et al., 2013). Joy et al. (2014) estimated the micronutrient deficiency risks due to inadequate intakes of seven minerals in Africa. They showed that deficiency risks were highest for calcium (54% of the population), followed by zinc (40%), selenium (28%), and iodine (19% after accounting for iodized salt consumption), while the risks for copper (1%) and magnesium (<1%) deficiencies were low (Joy et al., 2014). The deficiency risk for iron was lower than expected (5%), and multiple micronutrient deficiency risks were high in many countries (Joy et al., 2014).

While the world human population drastically increases, there is a corresponding reduction in availability of land for farming. To worsen this scenario, global warming has a deleterious impact on the agricultural productivity, with dire consequences on the food supply for both developed and developing countries (Rosenzweig and Parry, 1994). Africa, on the other hand, is a world region where a high diversity of food crops is grown. Vegetables and fruits are produced throughout the continent and are sources of much needed micronutrients. However, there is limited industrial scale processing of most of the agricultural products in the continent, leading to large economic losses of up to 40% and, as a consequence, to poverty and hunger (Gustavsson et al., 2011).

Africa is rich in the provision of traditional fermented foods, particularly those based on plant materials as substrates. These are often produced using minimal technology and inputs (Odunfa, 1985). Despite this, many people in sub-Saharan Africa are malnourished and this is due to agronomic constraints, as well as a lack of appropriate local food processing techniques. Accordingly, a huge proportion (ca. 30–50 %) of harvest is lost at the postharvest stage (Shiundu and Oniang’o, 2007). The main causes for this are inadequate production conditions (Abukutsa-Onyango, 2007), as well as rapid product decay during transport, storage, and marketing (Muchoki et al., 2007). Therefore, effective postharvest strategies based on sound scientific principles need to be developed for an efficient crop utilization. These should be applicable and adaptable to different situations in African countries, where there are varying levels of infrastructure and technology.

Traditional methods of processing and value addition to vegetables and fruits have a long history throughout Africa (Steinkraus, 1985). Odunfa (1985) identified food processing that involved fermentation as an important method to facilitate the availability of food and support food security throughout the continent. Cereals and tubers, as well as legumes, fruits, and vegetables are produced in large quantities in many parts of Africa, and because of their mostly perishable nature, these would be targets for optimized postharvest processing. Postharvest processing based on fermentation has been used to produce and increase the shelf life of a variety of foods at either household or small scale, cottage-type business in Africa for decades (Odunfa, 1985; Steinkraus, 1985). The many advantages of fermenting agricultural produce must have been recognized throughout the continent as important strategy for increasing micronutrient supply, improving palatability and detoxification, as well as shelf life and digestibility. The significance of food fermentation as a sustainable postharvest technology, especially for developing countries, has become well-recognized by FAO which published global perspectives (Battcock and Azam-Ali, 1998; Haard et al., 1999; Deshpande et al., 2000). Apart from contributing to the dietary intake of the people at both the macro- and micronutrient levels, it improves safety, quality and availability of foods and generates income for the food processors.

The aim of this review is to describe different lactic fermented fruit and vegetable fermentations that are currently utilized in Africa and to identify possible novel production processes. The involvement of the different microorganisms associated with the fermentations will be assessed. The beneficial roles that traditional fermented foods may play in the diet and health of African consumers will also be addressed, as well as the development of concepts that could facilitate development of new products or process optimization which may lead to products with improved safety, quality or added value.

Fruits and vegetables produced in the different regions of Africa are classified in this chapter as foods that include leafy vegetables, fruits, and protein-oil seeds. The starchy vegetables are not considered in this review. Very high percentages of fruits and vegetables are consumed after harvest in Africa. In many countries, traditional processing of fruits and vegetables play important roles in the food supply, especially during off seasons and harvest.

## Roles of Fruits and Vegetables in Nutrition and Health of African Consumers

Plant products including fruit and vegetables, cereals, legumes, seeds, roots, and tubers are an important source of fiber, carbohydrate, protein (**Table 1**), as well as source of amino acid, fatty acids, minerals (**Table 2**), and vitamins (**Table 3**). African leafy vegetables (ALVs) are a good source of vitamin A, being able to provide >75% of the recommended daily allowance (RDA; van Jaarsveld et al., 2014). Especially black nightshade, pigweed, cowpea and spider flower were found to have higher β-carotene content than conventional leafy vegetables. ALVs also have much higher mineral concentrations (>1% of plant dry weight) than conventional leafy vegetables, thus making them a superior source of mineral supplements (Odhav et al., 2007). Apart from this, they may also be an important source of antioxidants (Willcox et al., 2003). A shift in the oxidative potential in the human body has been recognized to be due to the limitation of antioxidants, which leads to oxidative stress and cellular oxidative damage. Antioxidants from fruits and vegetables were identified to be essential for the balancing of oxidative stress (Rautenbach et al., 2010) by way of supplying antioxidants such as vitamin C, carotenoids, tocopherols, and polyphenols, all which are important to human health.

**Table 1:** Proximate composition of some raw leafy African vegetables per 100 g fresh material.

	Moisture (g)	Protein (g)	Fat (g)	Total ash (g)	Dietary fiber (g)	Carbohydrates (g)
*Cucurbita maxima* (pumpkin leaves)^a^	87.3	4.24	0.12	3.23		
*Amaranthus tricolor* (misbredie)^a^	89.9	3.49	0.15	2.12		
*Corchorus tridens* (wild jute)^a^	81	5.19	0.25	3		

*Solanum retroflexum* (black nightshade)^b^	89.5	0.5	0.4	1.32	2.5	8.2
*Amaranthus cruentus* (pigweed)^b^	82	4.2	0.3	2.38	6.7	11.2
*Corchorus olitorius* (jew’s mallow)^b^	79.6	3.2	0.1	1.81	10.8	15.3
*Vigna ungui*culata (cowpea)^b^	82.4	4.7	0.6	1.76	5.8	10.5
*Cucurbita maxima* (pumpkin leaves)^b^	85.6	2.9	0.2	1.51	3	9.8
*Citrullus lanatus* (tsamma melon leaves)^b^	81.3	3.5	0.4	1.66	3.8	13.1
*Cleome gynandra* (spider flower)^b^	87.5	5	0.3	1.46	3.1	5.7

*Amaranthus hybridus* (cockscomb)^c^	85	6	0.5	4.91	2.81	6.09
*Bidens pilosa* (black jack)^c^	88	5	0.6	2.82	2.92	3.72

*^a^Schoenfeldt and Pretorius, 2011; ^b^van Jaarsveld et al., 2014; ^c^Odhav et al., 2007.*

**Table 2:** Mineral composition of some raw leafy African vegetables per 100 g fresh material.

	K (mg)	P (mg)	Ca (mg)	Mg (mg)	Mn (μg)	Fe (mg)	Cu (mg)	Zn (mg)
*Cucurbita maxima* (pumpkin leaves)^a^		119	383	142		15.9		0.9
*Amaranthus tricolor* (misbredie)^a^		70.6	232	141		16.2		0.8
*Corchorus tridens* (wild jute)^a^		136	585	80,9		6.3		0.8

*Solanum retroflexum* (black nightshade)^b^	257	36	199	92	2080	7.2	0.16	0.56
*Amaranthus cruentus* (pigweed)^b^	459	81	443	242	2340	5.1	0.17	0.7
*Corchorus olitorius* (jew’s mallow)^b^	407	118	310	87	790	3.6	0.19	0.57
*Vigna unguiculata* (cowpea)^b^	238	51	398	62	2690	4.7	0.14	0.42
*Cucurbita maxima* (pumpkin leaves)^b^	351	102	177	67	540	9.2	0.21	0.75
*Citrullus lanatus* (tsamma melon leaves)^b^	260	119	212	59	760	6.4	0.2	0.74
*Cleome gynandra* (spider flower)^b^	374	138	232	76	580	2.1	0.25	1.04

*Amaranthus hybridus* (cockscomb)^c^		106	401	224	4.1	4	0.3	3.1
*Bidens pilosa* (black jack)^c^		60	162	79	2.5	2	1.2	2.6

*^a^Schoenfeldt and Pretorius, 2011; ^b^van Jaarsveld et al., 2014; ^c^Odhav et al., 2007.*

**Table 3:** Selected vitamins of some raw leafy African vegetables per 100 g fresh material.

	Carotene (mg)	Vitamin A (μg) RAE	Ascorbic acid (mg)	B1 (mg)	B2 (mg)
*Cucurbita maxima* (pumpkin leaves)^a^	1.7				0.12
*Amaranthus tricolor* (misbredie)^a^	1.6				0.03
*Corchorus tridens* (wild jute)^a^	3.67				0.07

*Solanum retroflexum* (black nightshade)^b^	5.57	422	5	0.08	0.17
*Amaranthus cruentus* (pigweed)^b^	7.14	537	2	0.04	0.05
*Corchorus olitorius* (jew‘s mallow)^b^	4.3	329	1	0.02	0.03
*Vigna unguiculata* (cowpea)^b^	7.03	537	9	0.07	0.08
*Cucurbita maxima* (pumpkin leaves)^b^	4.25	325	2	0.04	0.1
*Citrullus lanatus* (tsamma melon leaves)^b^	4.96	375	10	0.01	0.1
*Cleome gynandra* (spider flower)^b^	5.94	434	2	0.06	0.21

*^a^Schoenfeldt and Pretorius, 2011; ^b^van Jaarsveld et al., 2014.*

Antioxidants play a role also in the prevention of development of chronic diseases such as cancer, cardio vascular disease (hypertension) and pathogenesis of immune deficiency virus (Willcox et al., 2003). Some fermented plant products have been shown to possess higher vitamin contents than the unfermented foods. This was the case for instance for fermented vegetable proteins occurring in fermentations for the production of *iru* or *dawadawa.* These contain higher levels of riboflavin than the unfermented seeds (Odunfa, 1986). Methionine- and lysine- producing lactobacilli strains have also been isolated from traditional fermented *ogi* (Odunfa et al., 2001). A novel *Lactobacillus rossiae* DSM15814^T^ species was shown to possess a complete *de novo* biosynthetic pathway for synthesis of riboflavin, vitamin B12 and other B vitamins (De Angelis et al., 2014), and an *in situ* study showed the relevance of such strains in cereal fermentations (Capozzi et al., 2012). Thus, in the fermentations the microorganisms or their products can contribute to the micronutrient supply and may thus contribute to prevention of malnutrition.

## Food Fermentation as a Postharvest Strategy for Food Security in Africa

Fermentation used as a traditional food processing technique, contributes to human energy food requirement, protein intake, fatty acids, and micronutrient intake. It has been well reported, that especially lactic acid fermentations used as traditional food processing techniques are based on general methods such as mechanical de-hulling of seeds, peeling of tubers, grating, boiling, soaking, and pressing the starting material in order to prepare the substrate for fermentation. This is followed then by the common fermentation stage, where microbial biochemical changes are brought about by wild-type lactic acid bacteria (LAB) that originate from the raw materials (Leroy and De Vuyst, 2004). These biochemical changes are based on the LAB sugar metabolism and result in product acidification, as well as a concomitant flavor enhancement and aroma development (Leroy and De Vuyst, 2004). Traditional processes that involve fermentation of agricultural products are common practice throughout Africa, with a long history of household and small scale, cottage-type level production (Kimaryo et al., 2000; Holzapfel, 2002). Many of the methods were developed based on a need for food preservation and for attaining an adequate nutrition (Nout and Motarjemi, 1997; Galati et al., 2014). Furthermore, fermentation processes resulted in acceptable developments of flavor and aromas, and/or in detoxification of product, which improve either the raw material sensory characteristics or render them edible (Holzapfel, 1997; Nout and Motarjemi, 1997).

Cereals (Nout, 2009; Franz et al., 2014; Galati et al., 2014) and starchy roots (Franz et al., 2014) are important substrates for probably the majority of African fermented plant products. This review, however, specifically addresses the fruit and vegetable fermentations in Africa, which are relatively less practiced and for which relatively less information is available. The major types of fruit and vegetable fermentations identified in different regions of Africa are classified here on the basis of LAB either dominating or occurring in co-metabolism with other microbes, thereby impacting biochemical transformation of different vegetal components. These include (i) lactic fermented leafy vegetables (ii) alkaline fermented vegetable proteins containing LAB (iii) fermented fruits. These will be discussed with different examples in the sections below. It should be noted that the classification of the bacteria associated with fermentations described in some of the older studies mentioned below were based on phenotypic and biochemical data only and may thus not be according to current classification.

## African Fermented Vegetables and Fruits

### Lactic Acid Fermented Leafy Vegetables

The tropical climate and agricultural land in Africa supports the growth of different leafy vegetables. Some ALV plants that are traditional to Africa and only successfully grow in this continent are listed in **Table 4**. Leafy vegetables have a short shelf life and are highly perishable, and different ALVs are indigenous to different regions of the continent (Shiundu and Oniang’o, 2007) (**Table 4**). Processing of ALVs immediately after harvest includes washing, shredding and drying. Sun-drying and fermentation are the two most important processing techniques used for processing of ALVs (Ayua and Omware, 2013). Some ALVs are also fermented after shredding, an example for this is the production of *kawal* in the Sudan, where the fresh leaves of the leguminous plant *Cassia obtusifolia* L. are fermented and they are consumed as meat or fish protein substitutes in soups and sauces (Suliman et al., 1987). The leaves are abundantly available and serve as cheap source of proteins and amino acids, with a high composition of oxalate (Dirar et al., 1985). Production of *kawal* involves a solid state fermentation of the leguminous leaves by bacterial species such as *Bacillus subtilis*, *Propionibacterium*, and *Staphylococcus sciuri*, with participation of LAB such as *L. plantarum* (Dirar et al., 1985).

**Table 4:** Distribution of some regional and common African leafy vegetables.

All over the sub-continent	West/East and Central Africa	West and Southern Africa	East/Central and Southern Africa
*Abelmoschus esculentus* (ladies’ fingers)	*Basella alba* (vine spinach)	*Amaranthus caudatus* (Aluma)	*Solanum nigrum* (black nightshade)
*Amaranthus cruentus* (amaranth)	*Citrullus lanatus* (watermelons)	*Amaranthus hybridus* (amaranth)	*Bidens pilosa* (black-jack)
*Corchorus olitorius* (jute mallow)	*Colocasia esculenta* (*cocoyam*)	*Portulaca oleracea* (purslane)	*Cleome gynandra* (African cabbage)
*Cucurbita maxima* (pumpkins)	*Hibiscus sabdariffa* (*zobo*)		
*Vigna unguiculata* (*cow-pea*)	*Ipomea batatas* (sweet potato)		
*Solanum macrocarpon* (African eggplant)	*Manihot esculenta* (cassava)		
	*Solanum aethiopicum* (mock tomato)		
	*Solanum scabrum* (garden huckleberry)		
	*Talinum triangulare* (waterleaf)		
	*Vernonia amygdalina* (ewuro)		
	*Moringa oleifera* (moringa or drumstick tree)		
	*Solanecio biafrae* (*Worowo*)		

*Adapted from Smith and Eyzaguirre (2007).*

In the Congo, *ntoba mbodi* is a fermented leafy vegetable consumed as condiment (Sanni and Oguntoyinbo, 2014). Kobawila et al. (2005) produced a flow diagram describing the fermentation processing of *ntoba mbadi*. The processing involves sun-drying cassava leaves for 2–3 h to wilt the leaves, which allows easier removal of stalks and petioles. The lamina are cut into fragments, washed with water, packed and wrapped in papaya (*Carica papaya* L.) leaves, and are then left to ferment for 2–4 days in a basket. The fermentation is a semi-solid process, alkaline fermentation, which leads to a steady increase in pH to 8.5. The bacteria reported to be involved include the *Bacillus* spp., *B. macerans*, *B. subtilis*, and *B. pumilus.* Other bacteria, such as *Staphylococcus xylosus* and *Erwinia* spp., as well as LAB such as *Enterococcus faecium*, *E. hirae*, *E. casseliflavus*, *Weissella confusa*, *Weisella cibaria*, and *Pediococcus* spp., have also been reported to co-occur in the fermentation (Ouoba et al., 2010; Sanni and Oguntoyinbo, 2014). It should be noted, that some of the bacteria mentioned above which occur in leafy vegetable fermentations are regarded as potentially pathogenic, as is the case for *Enterococcus* spp. such as *E. faecalis* and *E. faecium*, and for some toxinogenic *Bacillus* spp.

Apart from the effect of lactic preservative influence, reduction of cyanogenic acid in the leaves and mineralization, further beneficial changes are brought about by the fermentation process (Ouoba et al., 2010; Sanni and Oguntoyinbo, 2014). In Kenya, cowpea leaves (*Vigna unguiculata* syn. *Vigna sinensis*) are part of the diet, and a recent study showed that natural fermentation can improve the keeping quality, retaining β-carotene by 91% and ascorbic acid by 15%, while a sensory evaluation showed a good consumer acceptance of the fermented cowpeas (Muchoki et al., 2007). This study, as well as the study by Wafula et al. (2015), showed that cowpeas leaves do not contain sufficient levels of sugar to support the fermentation by autochthonous bacteria, and that sugar and preferentially also starter cultures should be added to obtain a reliable fermentation of this product.

In Kenya, African kale leaves are also processed in a fermentation-like manner, by soaking the vegetables in milk for a few days to achieve the removal of the bitter taste. However, little is known about the fermentation of kale and studies on which bacteria are important for the fermentation and on the dynamics of the fermentation are required.

### Alkaline Fermented Vegetable Proteins Involving Lactic Acid Bacteria in the Fermentation

A significant proportion of the protein intake in African countries is vegetal-plant-protein sources, notably the proteinaceous seeds (oil seeds), many of which are consumed in form of fermented vegetable proteins (Odunfa, 1988). The seeds bearing the cotyledon used in production of condiments are produced in large quantity in Africa, especially from members of the *Malvaceae* family plants, such as *Adansonia digitata, Parkia biglobosa*, *Prosopis africana, Hibiscus sabdariffa*, and from the *Fabaceae*, leguminous-bean producing plants, e.g., cowpeas (*Vigna unguiculata*) and soy beans (*Glycine max*; Parkouda et al., 2009). Some of African fermented vegetable proteinaceous seeds and the corresponding condiments produced and consumed from these in different regions of Africa are shown in **Table 5**.

**Table 5:** African fermented vegetable proteins with reported microorganisms involved.

Fermented food product	Country	Vegetal Substrate	Microorganisms	Reference
*Iru or Dawadawa*	Nigeria	*Pakia biglobosa*	*B. subtilis, B. amyloliquefaciens*, LAB	Adewumi et al., 2013
*Okpehe*	Nigeria	*Prosopis africana*	*B. subtilis, B. amyloliquefaciens, B. cereus*, and *B. licheniformis, Enterococcus* spp.	Oguntoyinbo et al., 2010
*Maari*	Burkina Faso	*Adansonia digitata*	*B. subtilis, E. faecium, E. casseliflavus, Pediococcus acidilactici*	Parkouda et al., 2009; Sanni and Oguntoyinbo, 2014
*Bikalga*	Burkina Faso		*B. subtilis, B. licheniformis, B. cereus, B. pumilus, B. badius, Weissella confusa, Weissella cibaria, L. plantarum, Pediococcus pentosaceus, Enterococcus casseliflavus, E. faecium, E. faecalis, E. avium, E. hirae, Brevibacillus bortelensis, B. Sphaericus*, and *B. fusiformis*.	Ouoba et al., 2008, 2010
*Ugba*	Nigeria	*Pentaclethra macrophylla*	*B. subtilis, B. licheniformis, B. megaterium, B. pumilus*	Anyanwu et al., 2016

The climatic condition in Africa favors a wide diversity and distribution of plants of the family *Malvaceae* across the continent. The seeds are, however, not directly consumed without processing, because of their anti-nutritional compounds such as proteinase inhibitors, amylase inhibitors, metal chelators, flatus factors, haemagglutinins, saponins, cyanogens, lathyrogens, tannins, allergens, acetylenic furan, and isoflavonoid phytoalexins (Pariza, 1996). *Parkia biglobosa* and soybean typically contain trypsin inhibitors, which reduce the digestibility of proteins (Collins and Sanders, 1976) and carbohydrate fractions that are responsible for flatulence after ingestion (Fleming, 1981). Soybean contains high levels (120–150 gkg^-1^ dry wt) of α-galactosides of sucrose, causing gastrointestinal gas production in humans (Sarkar et al., 1997). Kawamura (1954) observed that over 90% of the sugars present in ripe soybeans comprise sucrose and the indigestible (but fermentable) sugars raffinose and stachyose. Cottonseed also contains gossypol, an antinutritional factor, while mesquite seeds *Prosopis africana* can cause fetal abortion in domestic animals. However, there is long history of consumption of these seeds in Africa (Odunfa, 1985). Processing and fermentation must therefore have contributed significantly to the extensive hydrolysis of the seeds and concomitant detoxification. Different communities have developed strategies for processing of the seeds for food, especially through the use of natural fermentation, to produce foods which are rich in vegetable proteins and which are used as seasoning agents or as meat or fish substitutes (Odunfa, 1985; Steinkraus, 1996).

Traditional processing of these seeds includes wet de-hulling, boiling and fermentation. There are similar fermented vegetables proteins bearing different names in Africa, also the processing techniques often follow a similar methodology. The common examples of fermented vegetable proteins reported in Africa are shown in **Table 6**. The fermentation process during production has been described as an alkaline fermentation, due to the microbial enzymatic changes that involve hydrolysis of proteins to polypeptides, peptides, amino acids, and ammonia, thereby bringing about the increase in the pH value from 6.8 to 8.0. Fermented vegetable proteins have been described to be very rich in polyglutamic acid as a result of *Bacillus* metabolism, with compounds such as 3-hydroxybutanone (acetoin) and derivatives [butanedione (diacetyl) and 2,3-butanediol], acids (acetic, propanoic, 2-methylpropanoic, 2-methylbutanoic, and 3-methylbutanoic), as well as pyrazine also being produced.

**Table 6:** Examples of mixed lactic, acetic acid and alcoholic fermented vegetal starch beverages in Africa.

Fermented food product	Country	Vegetal Substrate	Microorganisms	Reference
*Tella*	Ethiopia	Sorghum	Yeast and LAB	Faparusi, 1973
*Burukutu*	Ethiopia Nigeria, Ghana	Guinea corn and cassava	*Saccharomyces cerevisiae*, *Lactobacillus plantarum* and *L. fermentum*	Faparusi, 1973
*Pito*	Nigeria, Ghana	Guinea corn and maize	*L. fermentum, L. delbrueckii, P. acidilactici*, *S. cerevisiae, C. tropicalis, K. apiculata, H. anomala, S. pombe, K. africanus*	Sefa-Dedeh et al., 1999; Sawadogo-Lingani et al., 2007
*Kaffir beer*	South Africa	Kaffir corn or maize	*Saccharomyces cerevisiae, Lactobacillus, Acetobacter*	Hesseltine, 1979; Odunfa and Oyewole, 1998
*Busaa*	East Africa	Maize	*Saccharomyces cerevisiae, Candida krusei, Lactobacillus plantarum, L. helveticus, L. salivarius, L. brevis, Weissella viridescens, Pediococcus damnosus, P. parvulus.*	Nout, 1980;
*Malawa beer*	Uganda	Maize	Unknown	–
*Zambian opaque beer*	Zambia	Maize	Unknown	–
*Merissa*	Sudan	Sorghum	LAB, yeast	Dirar, 1978
*Sekete*	Nigeria (south)	Maize	*A. aceti, A. pasteurianus, L. brevis, L. buchneri, L. plantarum, Lactobacillus* spp., *S. cerevisiae, Saccharomyces* spp., *Flavobacterium* spp., *Micrococcus varians, B. licheniformis*	Sanni et al., 1999
*Bouza*	Egypt	Wheat and maize	Unknown	
*Kishk*	Egypt	Wheat and milk	*Lactobacillus*, yeast, and *B. subtilis*	Morcos et al., 1973
*Tchoukoutou*	Benin	Sorghum	Yeast and LAB	Greppi et al., 2013

The ecology of microbes predominantly responsible for the important biochemical changes occurring during traditional fermentation of vegetal proteins was shown to involve diverse bacterial species. Starter cultures are generally not used, and natural fermentation is dominated by different bacteria with enzymatic activities, including *B. subtilis*-group bacteria such as *B. subtilis senso stricto*, *B. licheniformis, B. amyloliquefaciens*, and *B. pumilus*. In some alkaline fermentations of vegetal proteins, potentially pathogenic *B. cereus* strains were also described to occur (Oguntoyinbo et al., 2010). Studies indicated high proteolytic and amylolytic microbial activities, occurring from the onset of the fermentation for up to 48 h. Different species of LAB were also isolated during fermentation of vegetal proteins for condiment production in Africa. Ouoba et al. (2010) reported *Enterococcus faecium*, *E. hirae* and *Pediococcus acidilactici* to occur in *bikalga* and *soumbala.* Oguntoyinbo et al. (2007) isolated *Enterococcus* spp. from *okpehe* which led to a cheese-like aroma development during model fermentations, demonstrating that these bacteria could also affect the product characteristics in a negative way. As mentioned before, enterococci are not always regarded as favorable microorganisms because of the association of specific strains with infections in hospitals (Franz et al., 2011). A recent study showed that LAB could also play a positive role in the flavor development during fermentation of vegetable proteins of other legumes. An *in vitro* determination of volatile compound development during starter culture-controlled fermentation of *Cucurbitaceae* cotyledons showed that a mixed culture of *L. plantarum, Torulaspora delbrueckii*, and *Pediococcus acidilactici* could contribute to development of volatile compounds such as esters and low concentrations of aldehydes and ketones during fermentation (Kamda et al., 2015).

### Fermented Fruits

#### Alcoholic Beverages from Fruits Involving Lactic Acid Bacteria in the Fermentation

**FIGURE 1:**
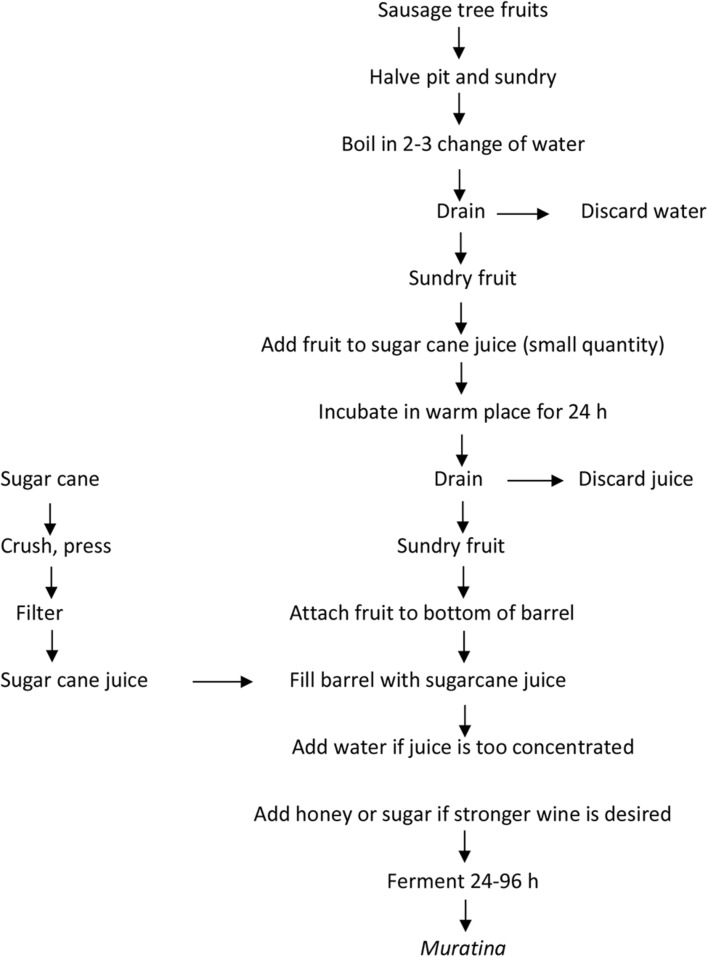
**Flow diagram of fermented sausage tree fruit for *muratina* production.** Adapted from Harkishor (1977).

Different fruits are grown in Africa and are harvested annually in different regions of the continent. Fruits used in Africa include banana, papaya, *marula*, mango, tomato, the sausage tree (*Kigelia Africana)* fruit and the *Ziziphus mauritiana* (*masau* or *jujube*) fruits. Because of the low pH and high acidity of fruits, microbial deterioration is very slow, and usually only osmophilic and acetotolerant microorganisms or yeasts are responsible for the major biochemical changes. Fruits are processed into different products that include juices, pickles, alcoholic beverages and vinegar. The fermentation aspect thus relies mostly on the production of alcoholic beverages or vinegars from fruit juices. A typical method of processing and fermentation of African fruit during *muratina* production from fruit of the ‘sausage tree’ (*Kigelia africana*) is shown in **Figure 1**. Other examples of fermented fruits in Africa are shown in **Table 7**. In Nigeria, information is available on *agadagidi*, an effervescent drink produced from ripe plantain (*Musa paradisiaca*) pulp. It is a popular drink in South Western Nigeria during ceremonies (Sanni and Oso, 1988; Sanni, 1989). Similarly, *uruaga* is a fermented banana in Uganda, while cashew and cocoa wine are also popular in Nigeria.

**Table 7:** Examples of mixed lactic, acetic acid and alcoholic fermented fruit beverages in Africa.

Fermented food product	Country	Fruit and vegetable	Fermentation	Microorganisms	Reference
*Agadagidi*	Nigeria	Plantain	Alcoholic	*Saccharomyces, Leuconostoc*, and *Streptococcus. Bacillus* and *Micrococcus*	Sanni, 1989
Cashew wine	Nigeria	Cashew	Alcoholic	Unknown	–
Cocoa wine	Nigeria	Cocoa	Alcoholic	Unknown	–
Palm wine *Emu* or *oguro*	Africa	Palm sap	Lactic, later alcoholic and acetic acid	*Lactobacillus plantarum, Leuconostoc mesenteroides, Fructobacillus durionis*, and *Streptococcus mitis.* Acetic acid bacteria. *Saccharomyces cerevisiae, Arthroascus, Issatchenkia, Candida, Trichosporon, Hanseniaspora, Kodamaea, Schizosaccharomyces, Trigonopsis*, *and Galactomyces.*	Faparusi and Bassir, 1972; Ehrmann et al., 2009; Ouoba et al., 2012
*Uruaga*	Kenya	Banana	Alcoholic and lactic	Unknown	–
*Ulansi*	East and South Africa	Bamboo	Alcholic and lactic	Unknown	–
*Muratina*	Kenya	*Sausage tree fruit (Kigelia africana)*	Alcholic and lactic	Uknown	–

Banana beer is a beverage popular throughout Africa and is made by fermenting banana juice with cereal flour, often sorghum flour (Marshall and Mejia, 2012). It is sweet and slightly hazy with a shelf life of several days. Ripe bananas (*Musa* spp.) are used, as these have high sugar content. Preparation involves extracting the juice from peeled bananas and the juice is diluted with clean, boiled water. Grinded cereal (sorghum or millet) is roasted over an open fire, added to the diluted banana juice in a bucket and left to ferment 18–24 h. The naturally occurring yeasts on the banana are responsible for fermentation. The ground cereal improves the color and flavor of the beer. After fermentation, the beer is filtered through a cotton cloth (Marshall and Mejia, 2012). In Rwanda, the banana beer ‘*urugwa*’ is produced by crushing and squeezing peeled ripe bananas to obtain juice that is then mixed with water to a desired proportion. Crushed roasted sorghum grains are added, and the mixture is then allowed to ferment for even 2–4 days in a warm pit covered with banana leaves (Shale et al., 2014). As banana beer is made from raw materials which are not boiled, the beer has only a short shelf life and should be kept as cool as possible, as it is an excellent substrate for microbial growth. Thus it is essential, that attention is paid to using clean equipment and processing area, as well as personal hygiene for the production of this beverage (Marshall and Mejia, 2012).

In Zimbabwe, wild fruits from the buffalo thorn (*Ziziphus mauritiana, masau)* are usually processed into porridge, traditional cakes, *mahewu* and jam (Nyanga et al., 2007). Moreover, they are also fermented to produce alcoholic beverages such as *kachasu*. They are crushed, soaked for some hours and then allowed to ferment (Gadaga et al., 1999). Nyanga et al. (2008) reported that *masau* is rich in citric-, tartaric-, malic-, succinic- and oxalic acids, as well as in minerals, fiber, crude protein and vitamin C (Nyanga et al., 2013). *Lactobacillus agilis, L. plantarum, W. minor, W. divergens, W. confusa, L. hilgardii, L. fermentum*, and *Streptococcus* spp. were isolated from *masau* fruit products and were identified as bacteria that could be developed as starter cultures for fermentation of the fruit products (Nyanga et al., 2007).

In Zimbabwe, an alcoholic beverage called *mudetemwa* is produced from the fruits of the sand apple (*Parinari curatellifolia*). The fruits are pounded and the juice is extracted by hand and boiled. After allowing to ferment overnight, the juice is again boiled, then allowed to cool and is drunk as beer (Gadaga et al., 1999). The fruit of the sugar plum tree (*Uapaca kirkiani*) are also used for production of alcoholic beverages in Zimbabwe. For this purpose, the fruits are pounded to break the skins and the seeds are extracted. The pulp is mixed with cold water and left to ferment into a sweet wine called *mutandavira* (Gadaga et al., 1999).

Recently, a fortified lactic acid fermented probiotic dairy product with a 14% (wt/vol) concentrated baobab fruit pulp, *mutandabota*, was developed in Zimbabwe. *Lactobacillus rhamnosus* (yoba) was used as starter culture for the fermented dairy drink, leading to a product with pH value of 3.5, which was rich in protein and vitamin C, with potential for improvement of intestinal health (Mpofu et al., 2014). The need for development of lactic fermented beverages that could support a healthy living and contribute to the dietary intake has been strongly suggested for the African population recently (Franz et al., 2014).

The fermented juice from palm sap of both *Rafia guineensis* and *Borassus akeassii*, popularly known as palm wine, is consumed widely in many African countries. During the fermentation process, *Saccharomyces cerevisiae* ferments the glucose as well as other plant derived carbohydrates such as sucrose, maltose and raffinose to produce alcohol. Apart from the yeasts, bacteria such as strains of *Leuconostoc*, *Lactobacillus*, and acetic acid bacteria have been described to play a role in the fermentation, and these were isolated at the initial and later stages of the fermentation, respectively (Amoa-Awua et al., 2007; Ouoba et al., 2012).

Wine is also produced from the fruits of the *marula* (*Sclerocarya birrea*) tree. A potent wine made from *marula* is *buganu*, which is produced in Swaziland (Simatende et al., 2015). For *buganu* production, fresh ripe fruits (10 kg) are washed and pounded or pressed to remove the juice. The juice, pulp and seeds are transferred to plastic buckets and water (10 L) is added, followed by the addition of 2 kg of sugar. The slurry is then fermented for 3 days at 25–30°C and additional water (10 L) is added. The mixture is then stirred and sieved with a traditional grass sieve or metal mash. Sugar (2 kg) is again added and the juice is fermented for a further 12 h at 25–30°C to obtain the *marula* wine *buganu* (Simatende et al., 2015). Both fermentative and non-fermentative yeasts were isolated from *marula* fruits, but the role of these in the production of *marula* wine has not been studied (Okagbue, 1995; Gadaga et al., 1999). Prouction of *marula* at a commercial scale has been achieved in South Africa, with the liquor Amarula, which is internationally available.

Fruit processing into wine is well developed at an industrial scale in South Africa. Grapes are commonly used for wine production, and LAB play an important role for instance in the malolactic fermentation important for biological de-acidification of wine. This is a decarboxylation process by which malic acid, a dicarboxylic acid naturally present in grape, is converted to lactic acid with concurrent liberation of carbon dioxide. This fermentation plays an important role in de-acidification and aroma development of specific wines. LAB such as *Oenococcus oeni*, and various species of *Lactobacillus* and *Pediococcus* have been reported to occur in wine or to play a role in malolactic fermentation during South African wine production (Du Toit et al., 2011; Miller et al., 2011). Recently, a bacteriocin-producing *Enterococcus faecium* was isolated from South African wine production, (Ndlovu et al., 2015), but whether such bacteria play a beneficial or detrimental role is currently not known.

#### Production of Vinegar from Fruit Juices

In Africa, different indigenous fruits are also processed into vinegar, however, at a very small scale. Fruit vinegars are made from fruit wines that are processed from fruits such as plum, mango, apple cider, *marula*, coconut and grapefruit (Ndoye et al., 2007). Ameyapoh et al. (2010) investigated the potential for vinegar production from mango (*Mangifera indica* var. Linn) in Togo. Vinegar was produced by a successive fermentation with *Saccharomyces cerevisiae* and acetic acid bacteria. For this, mangos were washed and peeled and mango juice was extracted by mechanical pressure. The juice was pasteurized and concentrated to obtain sugar content of 20° Brix. Yeasts were inoculated (2 mL, total no. of yeasts amounting to 10^6^ CFU) and the alcoholic fermentation was done at 30°C for 144 h (Ameyapoh et al., 2010). After this, acetic acid bacteria (2 mL, 10^6^ CFU total number bacteria) were added for the acetic acid fermentation at 30°C for 15 days. The successful fermentation in two stages led to a vinegar containing 4.7° acetic acid (mass in gram acetic acid in 100 g vinegar; Ameyapoh et al., 2010). This method for mango vinegar production may thus aid in avoiding postharvest losses, and can provide additional cash income for small-scale producers.

#### Effect of Fermentation on Detoxification and Nutrient Bioavailability

Fermentation is accompanied by a decomposition of macromolecules. Proteases are active during the alkaline fermentation of vegetable proteins, while amylases and pectinases are important in the macromolecule degradative processes of starchy vegetables. The enzymatic degradative processes result in the breakdown of proteins, carbohydrates and oligosaccharides and thus contribute to the release of important compounds essential to human nutritional requirements (Motarjemi and Nout, 1996). Processing by traditional fermentation thus relies on enzymes produced during germination or from bacteria during fermentation, and these contribute significantly to the bio-availability of macro- and micronutrients of fermented products. Microbial phytase activities may also contribute to the reduction of the antinutritive factor phytate, which occurs in various cereals and legumes (Kayode et al., 2007; Adeyemo and Onilude, 2014). The enzymatic activity of β-glucosidase enzymes of certain LAB or yeasts are important for the breakdown of cyanogenic glucosides such as linamarin and lotaustraline, which is present in maniok (*Manihot esculenta* var. Crantz; Okafor and Ejiofor, 1986; Kostinek et al., 2007) and this, combined with utilization of cyanhydric acid by certain *Bacillus* strains (Kobawila et al., 2005) significantly contribute to the detoxification of the final fermented products (Kostinek et al., 2007; Lambri et al., 2013).

From a health point of view, African vegetables and fruits contain significant levels of micronutrients, as well as high concentrations of bioactive compounds such as carotenoids, flavonoids, phenolic constituents, alkylresorcinols, glucosinolates and saponins which are present in many fruits and vegetables consumed in Africa and may contribute to the consumer’s health. Furthermore, the dietary fiber and vitamins in African fruits and vegetables, whose levels vary with cultivar, pre- and post-harvesting, processing and storage conditions (Nout, 2009; Uusiku et al., 2010; Medoua and Oldewage-Theron, 2011; Ogbuanu et al., 2014) are also relevant to consumer health. Microorganisms may play a pivotal role during fermentation in transforming chemical constituents, thereby enhancing the overall nutrition value of the final products via formation of health-promoting bioactive compounds, increased availability of vitamins and minerals, production of antimicrobial and antioxidant compounds or by stimulation of probiotic functions (Ðordević et al., 2010; Shahidi and Chandrasekara, 2013; Wang et al., 2014).

Yeast activity in the fermentation may also increase the vitamin content of vegetables and fruits, such as the availability of riboflavin, vitamin B12 and niacin. Riboflavin and niacin concentrations increased in alcoholic fermented vegetal starch products such as sorghum beer, a popular drink in South Africa, which has been shown to significantly reduce incidences of pellagra (Steinkraus, 2002). Palm wine is also a very rich source of ascorbic acid, thiamine and pyridoxine as well as vitamin B12 and other B vitamins (Steinkraus, 1997). Also, fermented foods are a rich source of folate, this compound is present in various green leafy vegetables, cereals, legumes and they have been linked to the prevention of heart disease, cancer and neuropsychiatric disorders (Brouwer et al., 1999). Group B vitamins (e.g., folic acid, riboflavin, thiamine, and cobalamin) are furthermore synthesized by a variety of LAB (LeBlanc et al., 2011). Vegetables and fruit products can become fortified with these vitamins, present in the biomass of LAB, as a result of fermentation. An increased content of niacin, thiamine, and riboflavin has thus been achieved through the fermentation of fluted pumpkin seeds (Achinewhu, 1986a), oil beans (Achinewhu, 1986b), and of melon seeds (Achinewhu and Ryley, 1987), to produce the condiments *iru* and *ogiri. Dawadawa*, which is also known as *uru*, *kpalugu*, *netetou*, or *soumbara*, is an African fermented food used as food condiment and meat substitute. It is obtained by fermentation of the African locust beans, which after fermentation have a higher digestibility than the unfermented beans, due to the enzymatic activity of the microbiota involved (Eka, 1980). *Dawadawa* contains a higher amount of riboflavin and thiamine as a result of fermentation, as well as a lower amount of flatus-forming oligosaccharides, the latter mainly due to the α- and β-galactosidase activities of the microbiota (Odunfa, 1983, 1985; Oboh et al., 2008).

## Research and Development Potential and Recommendations

The multiple problems that are still rampantly occurring on the African continent include problems of infrastructure, water supply, sanitation, and hygiene during processing. These, however, often still compromise the safety and quality of many traditional lactic fermented foods. Home and cottage sized, small-scale food processing endeavors, using crude techniques and rudimentary utensils, are mainly adopted and these are relatively uncontrolled processes, thereby exposing many of these foods to inconsistent quality or to different pathogenic microbes (Oguntoyinbo, 2014).

### Research and Development Potential

Processing using fermentation for value addition to fruits and vegetables is still majorly done in small scale and at household levels. Apart from supporting family nutritional intake, it also contributes to the economic activities, especially by increasing the income of women, who are the major processors and traders. Many of these fermented vegetal foods face safety or quality challenges and the strategies to ameliorate these challenges for sustainable industrial processing is further discussed.

#### Microbial Safety Challenges

Fermented vegetables and fruit face different microbial deterioration and safety issues. This is mainly a result of contamination during handling or post processing and cross contamination. Inadequate sanitation, inadequate and uninterrupted water supply and lack of good manufacturing practices are challenges to processors in developing countries. As mentioned above, potentially pathogenic bacteria such as *B. cereus* strains or *E. faecium* and *E. faecalis* strains have been described to occur as part of the microbiota of many vegetal protein or leafy vegetable fermentations. Different efforts and strategies have been suggested for the production of traditional vegetables and fruits in Africa, in order to guarantee microbial and chemical safety quality (Motarjemi and Nout, 1996; Holzapfel, 1997; Holzapfel, 2002). Development of Hazard Analysis and Critical Control (HACCP) is promising; it has been designed as base-line intervention strategy for some of the fermented vegetable protein such as *dawadawa* (Oguntoyinbo, 2012) and the fermented cassava product *fufu* (Obadina et al., 2009). Another strategy that has been proposed is the improvement on the back-slopping technique during fermentation. Back slopping refers to adding a small portion of a previous successful fermentation to a new fermentation, without knowing which microorganisms actually where present and responsible for the fermentation. For this improvement, an undefined mixture of starter cultures with known ability to dominate fermentations and more importantly, to inhibit pathogens is used as starting material to start fermentations. Starter cultures with such ability abilities have been selected in some pilot, as well as field studies for improvement of fermentation. Small portions of successful fermentation batches are kept and re-used for subsequent fermentation batches (Holzapfel, 2002). The fast growth of the starters and their success to establish themselves as dominant microorganisms in the fermentation leads to fast acidification and prevents growth of potential pathogens (Motarjemi and Nout, 1996; Holzapfel, 2002). An attractive alternative to back-slopping is the development of suitable starter cultures for fast growth and acidification in the fermentation medium (Holzapfel, 1997, 2002; Leroy and De Vuyst, 2004; Huch et al., 2008). However, for this a suitable industrial starter culture producer would need to be present locally, unless starter cultures are produced also at a household level using low-level technology (Holzapfel, 2002).

##### Process Optimization

Small scale traditional processing of vegetable and fruits is improving in term of scale-up technology. The processes now utilize specialized, mechanical equipment for grating and milling as well as fermentation tanks, cookers, and hydraulic presses. This has improved processing time and has aided in process scale-up. However, there is still a need for the development of techniques for larger scale industrialization, including peeling and de-hulling systems for seeds and tubers, pressure cookers and boilers, as well as industrial dryers. Optimized packaging and storage of fermented vegetables and fruits may also affect keeping quality and may improve attractiveness. There is ample opportunity for small business development in this sector, but this will depend on a close collaboration of small scale-producers with academic institutions who can provide the training in fermentation technology and who can develop and provide starter cultures. Food microbiologists and food technologists could work hand with women’s groups and local entrepreneurs, while local stakeholders and financial institutions could help to initiate small startup initiatives.

##### Nutritional Improvement

Nutritional value addition to fermented vegetal and fruits would contribute to the dietary status of consumers and thus toward a healthy population, and would also improve product acceptability. Such value addition may arise from the use of multifunctional starter cultures with high potential to increase the bio-availability of especially minerals, different vitamins and antioxidants. Thus, lactic fermentation could play an important role in the improvement of not only shelf life, but also the nutrient availability of fermented vegetal products. An open question which needs to be addressed is that of consumer acceptability of the local population. While lactic fermented foods are common in Africa, the fermentation of leafy vegetables is not common and studies would be required on the sensory acceptability of these products to local consumers.

##### Recommendations

The research and marketing potential for ALV fermentation should be given high priority. A high variety of indigenous vegetables rich in micronutrients occur in Africa and these should be utilized in order to minimize post-harvest losses. Fermentation is a likely post-harvest processing method that can prevent losses and which contributes to food security and safety. Fermentation of indigenous ALVs with selected starter cultures may lead to improved bio-availability and preservation of trace elements, vitamins and anti-oxidants. Advanced techniques for the production of locally fermented vegetables should be encouraged by local communities, local academic institutions, non-governmental organizations and other stakeholders. Age-old traditions of vegetable fermentation are typical for Europe (e.g., *sauerkraut*) and Asia (*kimchi* for Korea, and diverse vegetable fermentations on a household scale in China). These experiences may serve as valuable guidance for introducing similar (mainly lactic) and well-controlled, small-scale fermentations throughout the African continent, wherever leafy raw materials are available. Africa is rich in different leafy vegetables containing high amounts of nutrients and micronutrients (**Tables 1**–**4**). It is conceivable, therefore, that efforts for the fermentation of, e.g., cowpea, sorghum, spider plant, or kale leaves are intensified, in order to preserve the nutrients and prevent postharvest losses of such highly perishable products. What needs to be established, however, is whether leaves of these plants contain sufficient amounts of fermentable sugars to lend themselves for fermentation, or whether novel fermentation processes, based on selected starter cultures and on added fermentable sugars, need to be devised and tested. Lastly, it urgently needs to be established, if the local consumers agree to the taste of such fermented leafy vegetables. Sauerkraut may off course be a regional European food which appeals to people in the production region, but possibly not to the African taste. On the other hand, fermented products such as sorghum, cow pea or kale leaves probably don’t really taste like Sauerkraut and thus could be incorporated into local foods to agree to local tastes.

In addition, the potential for production of wines and vinegars from fruits should be intensified. Africa has a rich diversity of fruits in its gardens, which could be microbiologically enhanced to high quality vinegars or wines, as to obtain high value products. There certainly could be a good market in Africa or elsewhere for high quality, new juice products and vinegar products for example from indigenous fruits such as cactus pears, marula, Mobola plum (*Parinari curatellifolia*), wild loquat (*Uapaca kirkiana*), Dika tree fruit (*Irvingia barteri*), or wild orange (*Strychnos cocculoides*). There is much potential for fermentation of fruits and vegetables in Africa, what is needed is for universities and research institutes to work together with local producers and possibly NGO’s to help develop starter cultures, establish appropriate fermentation technologies, develop innovative and sustainable packaging and improve marketing of these local products.

## Author Contributions

FO, VF, CF, WH, AG, and HA wrote the main text regarding malnutrition, hidden hunger, and food processing in Africa. G-SC, WB, LF, and BT wrote the parts on nutrition contents and antioxidant activities of the vegetables. BB, JK, HN, NB, and MH wrote the parts on existing fermentations and improving the safety by fermentation, as well as the microbiology of the fermentations.

## Conflict of Interest Statement

The authors declare that the research was conducted in the absence of any commercial or financial relationships that could be construed as a potential conflict of interest.

## Footnotes

1 https://www.wfp.org/hunger/stats
